# The effect of exogenous glucagon-like peptide-1 on the glycaemic response to small intestinal nutrient in the critically ill: a randomised double-blind placebo-controlled cross over study

**DOI:** 10.1186/cc7874

**Published:** 2009-05-13

**Authors:** Adam M Deane, Marianne J Chapman, Robert JL Fraser, Carly M Burgstad, Laura K Besanko, Michael Horowitz

**Affiliations:** 1 University of Adelaide, Discipline of Anaesthesia and Intensive Care, North Terrace, Adelaide, 5000, South Australia, Australia; 2 Royal Adelaide Hospital, Department of Intensive Care, North Terrace, Adelaide, 5000 South Australia, Australia; 3 Investigation and Procedures Unit, Repatriation General Hospital, Daws Road, Daw Park, 5041, South Australia, Australia; 4 University of Adelaide, Discipline of Medicine, North Terrace, Adelaide, 5000 South Australia, Australia; 5 Royal Adelaide Hospital, Department of Gastroenterology, North Terrace, Adelaide, 5000 South Australia, Australia

## Abstract

**Introduction:**

Hyperglycaemia occurs frequently in the critically ill, affects outcome adversely, and is exacerbated by enteral feeding. Furthermore, treatment with insulin in this group is frequently complicated by hypoglycaemia. In healthy patients and those with type 2 diabetes, exogenous glucagon-like peptide-1 (GLP-1) decreases blood glucose by suppressing glucagon, stimulating insulin and slowing gastric emptying. Because the former effects are glucose-dependent, the use of GLP-1 is not associated with hypoglycaemia. The objective of this study was to establish if exogenous GLP-1 attenuates the glycaemic response to enteral nutrition in patients with critical illness induced hyperglycaemia.

**Methods:**

Seven mechanically ventilated critically ill patients, not previously known to have diabetes, received two intravenous infusions of GLP-1 (1.2 pmol/kg/min) and placebo (4% albumin) over 270 minutes. Infusions were administered on consecutive days in a randomised, double-blind fashion. On both days a mixed nutrient liquid was infused, via a post-pyloric feeding catheter, at a rate of 1.5 kcal/min between 30 and 270 minutes. Blood glucose and plasma GLP-1, insulin and glucagon concentrations were measured.

**Results:**

In all patients, exogenous GLP-1 infusion reduced the overall glycaemic response during enteral nutrient stimulation (AUC_30–270 min _GLP-1 (2077 ± 144 mmol/l min) vs placebo (2568 ± 208 mmol/l min); *P *= 0.02) and the peak blood glucose (GLP-1 (10.1 ± 0.7 mmol/l) vs placebo (12.7 ± 1.0 mmol/l); *P *< 0.01). The insulin/glucose ratio at 270 minutes was increased with GLP-1 infusion (GLP-1 (9.1 ± 2.7) vs. placebo (5.8 ± 1.8); *P *= 0.02) but there was no difference in absolute insulin concentrations. There was a transient, non-sustained, reduction in plasma glucagon concentrations during GLP-1 infusion (t = 30 minutes GLP-1 (90 ± 12 pmol/ml) vs. placebo (104 ± 10 pmol/ml); *P *< 0.01).

**Conclusions:**

Acute, exogenous GLP-1 infusion markedly attenuates the glycaemic response to enteral nutrition in the critically ill. These observations suggest that GLP-1 and/or its analogues have the potential to manage hyperglycaemia in the critically ill.

**Trial Registration:**

Australian New Zealand Clinical Trials Registry number: ACTRN12609000093280.

## Introduction

Hyperglycaemia occurs frequently, even in patients without pre-existing diabetes [1], and adversely affects outcome [2]. For this reason, treatment with insulin is widely used; however, insulin therapy is associated with a substantial risk of hypoglycaemia, which is associated with both short- and long-term adverse events [3,4]. Although the use of parenteral nutrition affords a stable caloric load, which minimises the incidence of hypoglycaemia [2], enteral feeding is the preferred method of nutrient delivery in critically ill patients [5]. Hence, there is a need for a therapy to manage hyperglycaemia in enterally fed patients without the risk of hypoglycaemia [6].

Exogenous administration of the incretin hormone, glucagon-like peptide-1 (GLP-1), has been shown to normalise blood glucose concentrations both in healthy patients and those with type 2 diabetes [7]. This occurs as a result of stimulation of insulin secretion, suppression of glucagon release and slowing of gastric emptying [7,8]. Because the former effects are glucose dependent, the use of GLP-1 does not appear to be associated with hypoglycaemia [9]. The effect of GLP-1 on glycaemia in enterally fed critically ill patients has hitherto not been evaluated. The primary aim of this study was to determine whether exogenous GLP-1 attenuates the glycaemic response to small intestinal nutrient infusion in critically ill patients not previously known to be diabetic.

## Materials and methods

### Subjects

Seven critically ill adult patients (four males, three females, age range 28 to 76 years), predicted to remain mechanically ventilated for more than 48 hours, were studied. Exclusion criteria included pregnancy, pre-existing diabetes, contraindication to enteral feeding or post-pyloric catheter insertion, and previous surgery on the oesophagus, stomach or duodenum.

The study was approved by the Human Ethics Committee of the Royal Adelaide Hospital and performed according to the Australian National Health and Medical Research Committee guidelines for the conduct of research involving unconscious persons. Written, informed consent was obtained from the next of kin.

### Study protocol

Patients were studied on two consecutive days, in which they received intravenous GLP-1 or placebo in a randomised, double-blind fashion. Twelve hours prior to the study, a naso-duodenal feeding catheter was inserted and confirmed via abdominal x-ray. On each study day enteral feeding was ceased at least four hours prior to the commencement of the study. Exogenous insulin (Actrapid, Novo-Nordisk, Copenhagen, Denmark) infusion was ceased at least two hours before the commencement of the study drug. Patient weight was provided by a relative and/or estimated by a dietician. Randomisation and reconstitution of synthetic GLP-1-(7–36) amide (Merck Biosciences, Melbourne, Australia), as a solution in 4% albumin, was performed by the Royal Adelaide Hospital Pharmacy department. The study investigators were blinded to each infusion, which were concealed in a glass bottle covered by black plastic. Both GLP-1 (1.2 pmol/kg/min) and placebo (4% albumin) were infused at a rate of 1 ml/min via a central venous catheter for 270 minutes (i.e. t = 0 to 270 min). At t = 30 minute Ensure^® ^(Abbott, Melbourne, Australia), a mixed nutrient liquid (64% carbohydrate, 1 kcal/ml), was delivered continuously into the small intestine at a rate of 1.5 ml/min for four hours (i.e. t = 30 to 270 min). An arterial blood sample was obtained every 15 minutes for measurement of blood glucose and at timed intervals for measurements of plasma insulin, GLP-1 and glucagon concentrations.

### Data analysis

Blood glucose was measured at the bedside using a portable glucometer (Medisense Optimum, Abbott, Melbourne, Australia). Blood was collected, separated by centrifugation and the resulting plasma was stored at -70° until assayed for hormone concentrations. Plasma insulin was measured by ELISA (Diagnostics Systems Laboratories, Webster, Texas, USA) with an inter-assay coefficient of variation (CV) of 6.2%. Total plasma GLP-1 (GLPIT-36HK Linco Research, St. Charles, Missouri, USA) and glucagon (Siemens Medical Solution Diagnostics, Berkeley, California, USA) concentrations were measured by radioimmunoassay, with a CV of 9.2% and 12%, respectively.

### Statistical analysis

Data are presented as mean ± standard error of the mean. Area under the curve (AUC) was calculated using the trapezoidal rule. The number of patients required to establish a glucose lowering effect of GLP-1 was based on power calculations derived from our previous work [8]. The ratios of insulin/glucose were calculated, as described previously [10]. Statistical analyses were performed using SPSS (Version 15.0, Chicago, Illinois, USA). Distribution and sensitivity analysis, using nonparametric analyses, allowed parametric testing of data. The difference between intervention and placebo was assessed using paired samples; Student's paired t-test and repeated measures analysis of variance. Data were evaluated for potential carry over effect. The null hypothesis was rejected at the 0.05 significance.

## Results

The study was well tolerated in all patients. Patient details are shown in Table 1.

**Table 1:** Summary demographic data of patients studied

Age	Mean 58 years
Sex	4 male:3 female
Diagnosis	4 medical:3 surgical
APACHE II (admission)	Mean 18
APACHE II (study)	Mean 17
Days in intensive care unit	Mean 7 days
Serum creatinine (μmol/l)	Mean 73 μmol
Plasma albumin(g/l)	Mean 23 g/l
Feed tolerant^1^	5 of 6 tolerant of EN
Inotropes	1 patient on catecholamine infusion
Parenteral nutrition	0 of 7
Weight	Mean 84 kgs
Rate of insulin infusion when ceased^2^	Mean 2.0 U/hour
Total insulin dose in previous 24 hours	Mean 57 U/24 hours

All data refer to results from the first study day unless otherwise specified.

^1 ^Patients are feed-tolerant if (over the preceding 24 hours): delivery of nutrient liquid is at the target rate (as determined by on-site dietician) and gastric residual volumes less were than 250 mL in any six-hour period.

^2 ^The infusion rate at t = -120 minutes (i.e. when exogenous insulin ceased).

APACHE = Acute Physiology and Chronic Health Evaluation.

### Blood glucose

**Figure 1:**
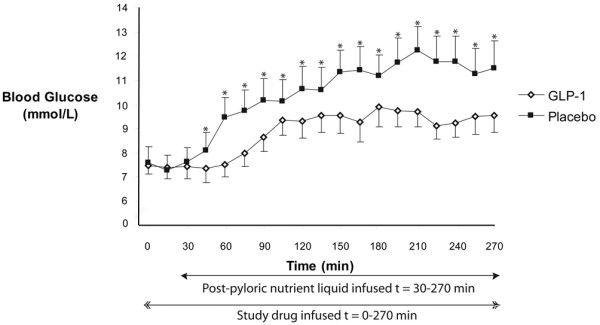
Exogenous glucagon-like peptide-1 (GLP-1) attenuated the rise in blood glucose levels and the overall glycaemic response to intra-duodenal nutrient infusion. (AUC_30–270 min _GLP-1 2077 ± 144 mmol/l min vs. placebo 2568 ± 208 mmol/l min; *P *= 0.02). Data are mean ± SEM (n = 7). * *P *< 0.05.

Blood glucose concentrations are shown in Figure 1. There was no difference in baseline blood glucose concentration prior to each infusion (t = 0 min GLP-1 7.5 ± 0.4 mmol/l vs. placebo 7.6 ± 0.6 mmol/l; *P *= not significant (NS)). Prior to the commencement of the small intestinal nutrient infusion (t = 30 min) GLP-1 had no effect on blood glucose. On both days, there was an increase in blood glucose concentration in response to intra-duodenal nutrient infusion (t = 180 min GLP-1 9.3 ± 0.6 mmol/l; placebo 12.2 ± 0.9 mmol/l; *P *< 0.01 for both). GLP-1 markedly attenuated the rise in blood glucose (e.g. t = 60 min GLP-1 7.5 ± -0.5 mmol vs. placebo 9.5 ± -0.8 mmol; *P *< 0.01), peak blood glucose (GLP-1 10.1 ± 0.7 mmol/l vs. placebo 12.7 ± 1.0 mmol/l; *P *< 0.01) and decreased the overall glycaemic response (AUC_30–270 min _GLP-1 2077 ± 144 mmol/l min vs. placebo 2568 ± 208 mmol/l min; *P *= 0.02).

### Plasma insulin

**Figure 2:**
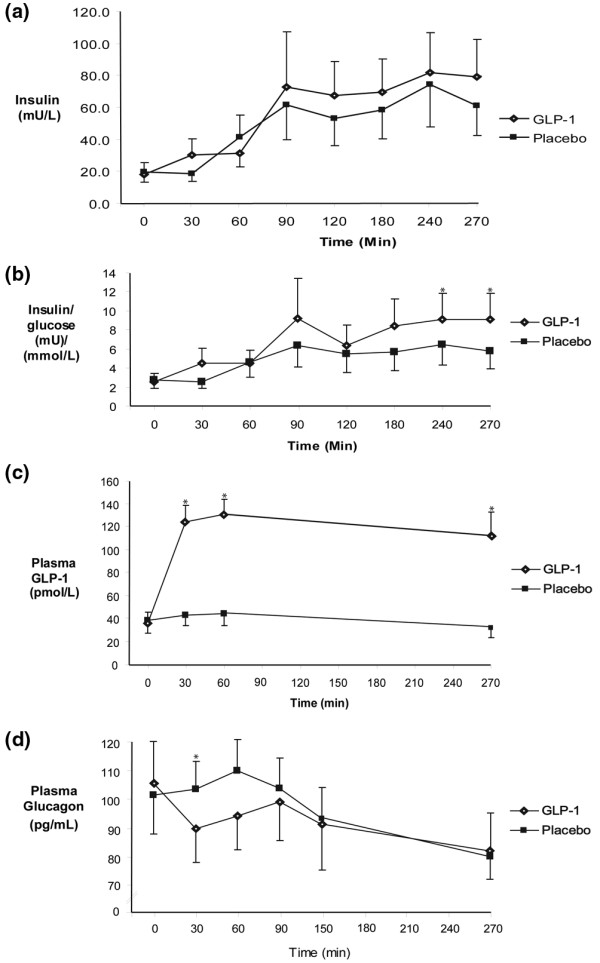
Plasma Hormone concentrations. There was no increase in total postprandial insulin secretion **(a)**, however the plasma insulin/blood glucose ratio was increased at t = 270 minutes **(b)**. Exogenous glucagon-like peptide-1 (GLP-1) infusion increased plasma GLP-1 concentrations **(c) **and caused a transient, but non-sustained, suppression of glucagon **(d)**. Data are mean ± SEM (n = 7). * *P *< 0.05.

Plasma insulin and insulin:glucose ratio is shown in Figures 2a and 2b. There was no difference in plasma insulin concentration at baseline. Plasma insulin increased in response to intra-duodenal nutrient (e.g. t = 270 min GLP-1 79 ± 21 mU/l and placebo 61 ± 17 mU/l; *P *< 0.03 compared with fasting concentration for both days). At 270 minutes, the insulin/glucose ratio was greater with GLP-1 (GLP-1 9.1 ± 2.7 vs. placebo 5.8 ± 1.8; *P *= 0.02); however, there was no difference in absolute plasma insulin concentrations throughout the entire study period (AUC_0–270 min _GLP-1 16,203 ± 5193 mU/l min vs. placebo 14,434 ± 4561 mU/l min; *P *= NS).

### Plasma GLP-1

Plasma GLP-1 concentrations are shown in Figure 2c. Fasting plasma GLP-1 concentrations were similar between groups (t = 0 min GLP-1 36 ± 10 pmol/l vs. placebo 38 ± 11 pmol/l; *P *= NS). Exogenous GLP-1 markedly increased plasma GLP-1 concentration within 30 minutes (t = 30 min GLP-1 124 ± 15 pmol/l vs. placebo 43 ± 9 pmol/l; *P *< 0.01) and throughout the infusion period (AUC_0–270 min _GLP-1 31,659 ± 4203 pmol/l min) vs. placebo 10,399 ± 2508 pmol/l min; *P *< 0.01). During GLP-1 infusion, steady state concentrations were achieved after 30 minutes (t = 30 min plasma GLP-1 124 ± 15 pmol/l vs. t = 90 min plasma GLP-1 131 ± 13 pmol/l; *P *= NS).

### Plasma glucagon

Plasma glucagon concentrations are shown in Figure 2d. Fasting glucagon concentrations were similar between study days (t = 0 min GLP-1 106 ± 14 pmol/ml vs. placebo 102 ± 13 pmol/ml; *P *= NS). There was a decrease in plasma glucagon from baseline during GLP-1 infusion (t = 30 min GLP-1 90 ± 12 pmol/ml vs. placebo 104 ± 10 pmol/ml; *P *< 0.01), which was non-sustained (AUC_0–270 minutes _GLP-1 22,786 ± 6040 pmol/l min vs. placebo 25,830 ± 2644 pmol/l min); *P *= NS).

## Discussion

This is the first study to evaluate the effect of exogenous GLP-1 infusion on the glycaemic response to enteral nutrition during critical illness. Given the need to avoid hyperglycaemia and hypoglycaemia in critically ill patients, an assessment of the effect of exogenous GLP-1 is of considerable interest.

The dose of GLP-1 used in the current study was based on a previous study in which infusion of GLP-1 at 1.2 pmol/kg/min achieved fasting normoglycaemia, and was well tolerated in postoperative patients with type 2 diabetes [11]. Hyperglycaemia in critically ill patients, not previously known to have diabetes, is associated with poorer outcomes than in patients with pre-existing diabetes [12]. Hence, we chose to study the effect of exogenous GLP-1 in these patients. Insulin infusions were ceased two hours before the commencement of the study to ensure clearance of exogenous insulin. Given the short plasma half life of GLP-1 [13] carry over effects were not anticipated or observed, and a crossover protocol was an appropriate study design. The nutrient type and load were selected on a feeding regimen which aims to deliver 25 kcal/kg/day of mixed nutrient liquid [14]. Accordingly, 1.5 kcal/min of Ensure^® ^was administered. Hyperglycaemia in critical illness is believed to reflect inadequate insulin secretion, hepatic and peripheral insulin resistance, and an increase in the counter-regulatory hormones cortisol, catecholamines, glucagon and growth hormone [15]. Only plasma insulin and glucagon concentrations were measured, as GLP-1 is not known to alter the secretion of the other counter-regulatory hormones and the study was designed to establish proof of concept [9].

Exogenous GLP-1 slows gastric emptying substantially [8] and this may be the dominant mechanism by which GLP-1 reduces postprandial glycaemic excursions [16]. Hence, the magnitude of glucose lowering by GLP-1 is likely to be even greater during gastric, rather than small intestinal, nutrient administration [17]. However, delayed gastric emptying occurs in approximately 50% of critically ill patients and, when marked, may lead to under-nutrition, gastro-oesophageal reflux and pulmonary aspiration [18]. Given the above considerations it was appropriate to initially determine whether GLP-1 attenuates the glycaemic response to small intestinal, rather than intra-gastric, nutrient. Whether GLP-1 will slow gastric emptying further in critically ill patients remains to be determined.

It should be recognised that nutrient-induced hyperglycaemia was only attenuated, and not suppressed completely by GLP-1. This may potentially reflect an insufficient GLP-1 dose, and larger doses are known to be well tolerated [19]. As discussed, given the effect of GLP-1, to slow gastric emptying, it is possible that the magnitude of glucose lowering will be greater during gastric feeding. However, some critically ill patients may also have inadequate β-cell reserve to compensate for the disordered hormone milieu even at larger doses and/or during gastric feeding. Given the complexity and severity of disordered glucose metabolism in the critically ill, and an ongoing requirement for nutrition, it is anticipated that exogenous GLP-1 may achieve normoglycaemia only in specific sub-groups of patients. However, this study establishes the concept that GLP-1 as sole therapy, or in combination with insulin, has the potential to manage hyperglycaemia in the critically ill.

We elected to evaluate the effects of an acute GLP-1 infusion in a relatively small, heterogeneous cohort of critically ill patients studied at various times after their admission. This limitation should be recognised and may have been of greater relevance if the study outcome had been negative, rather than positive. Although our study only measured the effect of a short-term infusion, it is likely that the glucose-lowering effect would persist for the entire period of GLP-1 administration [20]. Furthermore, we speculate that longer-term GLP-1 infusion may result in less glycaemic variability than the current approach to insulin therapy, because of its effects on both insulin and glucagon. An additional limitation is the inconclusive evidence regarding insulin stimulation and glucagon suppression. Although there were no observed differences in absolute insulin concentrations during the entire GLP-1 infusion, there was an increase in the insulin/glucose ratio and transient suppression of glucagon secretion. Moreover, the number of subjects included was based on power calculations for a glucose-lowering effect and the study may have been underpowered to establish effects on insulin and glucagon over the entire study period. Given the positive outcome of this study, additional studies are required to further elucidate the mechanisms underlying the effects of GLP-1 and determine the optimal dose and duration of treatment in the critically ill.

## Conclusions

This study establishes that exogenous GLP-1 infusion limits the peak blood glucose, and markedly attenuates the overall glycaemic response, during small intestinal feeding, in non-diabetic critically ill patients. Given exogenous GLP-1 may improve the safety and efficacy of glycaemic control in this group, further investigation into its potential use is warranted.

## Key messages

• The effects of exogenous GLP-1 are glucose dependent, thus the use of GLP-1 is not associated with hypoglycaemia.

• Exogenous GLP-1 markedly attenuates the glycaemic response to small intestinal nutrient in critically ill patients.

• Exogenous GLP-1 is a novel therapy to treat hyperglycaemia and further investigation into its potential use in the critically ill is warranted.

## Abbreviations

AUC: area under the curve; CV: coefficient of variation; ELISA: enzyme linked immunosorbent assay; GLP-1: glucagon-like peptide-1; NS: not significant.

## Competing interests

The authors declare that they have no competing interests.

## Authors' contributions

AD was the main contributor to study design, acquisition, analysis and interpretation of the data and drafting the manuscript. MJC and RF contributed to study conception and revision of manuscript. CB and LKB were responsible for data acquisition and analysis and contributed to revision of manuscript. MH was the main contributor to study conception and participated in drafting the manuscript. All authors read and approved the final manuscript.

## Authors' information

AD is an intensivist enrolled as a PhD student at the University of Adelaide. His thesis studies the effects of incretin hormones in critically ill patients. He is supervised by MJC, RJF and MH. The results were presented in abstract form at the 2008 meeting of the Australian New Zealand Intensive Care Society.
